# Novel 3,4-Dihydroisocoumarins Inhibit Human P-gp and BCRP in Multidrug Resistant Tumors and Demonstrate Substrate Inhibition of Yeast Pdr5

**DOI:** 10.3389/fphar.2019.00400

**Published:** 2019-04-16

**Authors:** Julia Sachs, Katja Döhl, Anja Weber, Michele Bonus, Ferdinand Ehlers, Edmond Fleischer, Anette Klinger, Holger Gohlke, Jörg Pietruszka, Lutz Schmitt, Nicole Teusch

**Affiliations:** ^1^Bio-Pharmaceutical Chemistry and Molecular Pharmacology, Faculty of Applied Natural Sciences, Technische Hochschule Köln, Leverkusen, Germany; ^2^Institute of Biochemistry, Heinrich-Heine-Universität Düsseldorf, Düsseldorf, Germany; ^3^Institute of Bioorganic Chemistry, Heinrich-Heine-Universität Düsseldorf im Forschungszentrum Jülich, Jülich, Germany; ^4^Institute for Pharmaceutical and Medicinal Chemistry, Heinrich-Heine-Universität Düsseldorf, Düsseldorf, Germany; ^5^MicroCombiChem GmbH, Wiesbaden, Germany; ^6^John von Neumann Institute for Computing, Jülich Supercomputing Centre and Institute for Complex Systems – Structural Biochemistry, Forschungszentrum Jülich GmbH, Jülich, Germany; ^7^IBG-1: Biotechnology, Forschungszentrum Jülich, Jülich, Germany

**Keywords:** multidrug resistance, cancer chemotherapy, 3, 4-dihydroisocoumarin, P-glycoprotein, breast cancer resistance protein, Pdr5

## Abstract

Multidrug resistance (MDR) in tumors and pathogens remains a major problem in the efficacious treatment of patients by reduction of therapy options and subsequent treatment failure. Various mechanisms are described to be involved in the development of MDR with overexpression of ATP-binding cassette (ABC) transporters reflecting the most extensively studied. These membrane transporters translocate a wide variety of substrates utilizing energy from ATP hydrolysis leading to decreased intracellular drug accumulation and impaired drug efficacy. One treatment strategy might be inhibition of transporter-mediated efflux by small molecules. Isocoumarins and 3,4-dihydroisocoumarins are a large group of natural products derived from various sources with great structural and functional variety, but have so far not been in the focus as potential MDR reversing agents. Thus, three natural products and nine novel 3,4-dihydroisocoumarins were designed and analyzed regarding cytotoxicity induction and inhibition of human ABC transporters P-glycoprotein (P-gp), multidrug resistance-associated protein 1 (MRP1) and breast cancer resistance protein (BCRP) in a variety of human cancer cell lines as well as the yeast ABC transporter Pdr5 in *Saccharomyces cerevisiae*. Dual inhibitors of P-gp and BCRP and inhibitors of Pdr5 were identified, and distinct structure-activity relationships for transporter inhibition were revealed. The strongest inhibitor of P-gp and BCRP, which inhibited the transporters up to 80 to 90% compared to the respective positive controls, demonstrated the ability to reverse chemotherapy resistance in resistant cancer cell lines up to 5.6-fold. In the case of Pdr5, inhibitors were identified that prevented substrate transport and/or ATPase activity with IC_50_ values in the low micromolar range. However, cell toxicity was not observed. Molecular docking of the test compounds to P-gp revealed that differences in inhibition capacity were based on different binding affinities to the transporter. Thus, these small molecules provide novel lead structures for further optimization.

## Introduction

Cancer remains the second most common cause of death worldwide with 8.7 million deaths in 2015 (Fitzmaurice et al., 2017). Despite constant progress in antitumor drug development, multidrug resistance (MDR) poses a major problem in effective patient treatment. MDR is estimated to cause treatment failure in about 90% of patients with recurrent tumors (Longley and Johnston, 2005). Additionally, it becomes a more and more severe problem in the treatment of pathogens. In general, MDR encompasses intrinsic or acquired resistance of cancer cells or pathogens to a spectrum of drugs, finally leading to reduction of treatment options and to therapy failure. Several mechanisms mediating MDR have been described, including mutations in drug targets, alterations in drug metabolism, decreased uptake or increased efflux of the drug (Kaye and Kaye, 2000; Gillet and Gottesman, 2010).

Probably the most prominent and widespread mechanism in eukaryotic cells is covered by increased drug transport from the cytoplasm through overexpression of ATP-binding cassette (ABC) transporters (El-Awady et al., 2016). The ABC transporter superfamily, representing one of the oldest and largest protein families, is expressed from archaea to human. The common mechanism for all ABC transporters encompasses the active membrane translocation of a broad spectrum of substrates using energy from ATP hydrolysis (Szöllõsi et al., 2017).

Regarding MDR development accompanying antitumor therapy, three ABC transporters were identified as the main contributors in humans. P-glycoprotein (P-gp, ABCB1), multidrug resistance-associated protein 1 (MRP1, ABCC1) and breast cancer resistance protein (BCRP, ABCG2) are frequently overexpressed in chemotherapy-resistant tumors, where they facilitate resistance to wide varieties of drugs commonly used in clinical practice (Bugde et al., 2017). Furthermore, recent studies have shown a pronounced influence of ABC transporters in drug resistance mechanisms controlled by cancer stem cells, mainly responsible for recurrence of the disease (Begicevic and Falasca, 2017).

The ABC transporter Pdr5 from *Saccharomyces cerevisiae* is located in the plasma membrane (PM) and acts in combination with other exporter pumps as a first line of defense against structurally unrelated xenobiotic compounds forming the pleitropic drug resistance (PDR) network (Ernst et al., 2005). Pdr5 is the most abundant exporter pump in yeast and highly homologous to multidrug mediating transporters of clinical relevant fungi such as *Candida albicans*. Each year billions of people become infected with pathogenic fungi and 1.5 million people are killed because of that (Brown et al., 2012). One of the largest problems is the evolving resistance shift of several fungi against azoles, which prevent one of the most efficient treatments. Therefore, the development of new antifungal drugs is of great importance.

Since the discovery of ABC transporter and characterization of their significant contribution to MDR, pharmacological strategies to overcome transporter-mediated MDR have been in the focus of various drug discovery approaches (Szakács et al., 2006; Tillotson and Theriault, 2013). One focus is the development of small molecule inhibitors interfering with transporter activity in combination with chemotherapeutic drugs, thereby increasing intracellular drug accumulation and efficacy by reversing resistance (Zhang and Ma, 2010; Spengler et al., 2017; Tran-Nguyen et al., 2017). ABC transporters involved in MDR often have overlapping substrate spectra. For example, several tyrosine kinase inhibitors including dasatinib and imatinib were demonstrated to become translocated to the extracellular space by P-gp and BCRP (Dohse et al., 2010). This leads to reduced efficacy of those drugs and subsequent therapy failure.

In most tumors, MDR is not only mediated by overexpression of one ABC transporter, but by expression of several transporters. Furthermore, P-gp and BCRP are co-expressed at the blood–brain barrier and prevent effective treatment of brain tumors (Agarwal et al., 2011). Hence, the design of dual transporter inhibitors appears to be more efficacious in mentioned cases. To date, several dual inhibitors of P-gp and BCRP have been identified including tariquidar and derivatives, aurones and chalcones (Sim et al., 2011; Gu et al., 2014; Li et al., 2015). Tariquidar entered clinical trials up to phase II to circumvent P-gp mediated MDR, but did not reveal sufficient clinical activity (Pusztai et al., 2005).

Natural compounds and their derivatives play an important role in drug discovery and development (Newman and Cragg, 2016; Guo, 2017). In this context, various ABC transporter inhibitors from natural sources were identified and characterized in the last decades (Karthikeyan and Hoti, 2015). Early natural product P-gp inhibitors such as cyclosporine A or its synthetic derivative PSC833 were tested in clinical trials up to phase III, but failed due to toxicity or other unintended side effects (Warner et al., 1995; Baer et al., 2002). Among them were several dual inhibitors of P-gp and BCRP, for example some aurones, chalcones or flavonoids (Boumendjel et al., 2009; Sim et al., 2011; Yuan et al., 2012; Iriti et al., 2017).

Isocoumarins, isomers of coumarin, represent a large class of secondary metabolites, which can be found in bacteria, fungi, lichens, marine sponges and to a lesser extent in higher plants (Saeed, 2016). To date, several 100 different isocoumarins and dihydroisocoumarins from nature have been identified and derivatives have been synthesized. Owing to their great structural diversity, diverse biological and pharmacological activities of isocoumarins including cytotoxicity and antimetastatic effects against various cancer types including breast, colon, melanoma (Cichewicz et al., 2004; Abid et al., 2012; Guimarães et al., 2016; Zhang et al., 2017), inhibition of inflammation (Liang et al., 2011; Ramanan et al., 2016) or different enzymes including aromatase and kallikrein peptidases as potential cancer targets (Endringer et al., 2008; Teixeira et al., 2011) as well as antibacterial, antifungal and antimalarial activities (Hoepfner et al., 2012; Lai et al., 2016; Simic et al., 2016) could be identified. Several natural or synthetic coumarins were identified as inhibitors of either P-gp, BCRP or both, yet activities were mostly moderate, and compound concentrations between 10 and 100 μM had to be applied for transporter inhibition (Barthomeuf et al., 2005; Raad et al., 2006; Combes et al., 2011; Bisi et al., 2017; Sjöstedt et al., 2017). In contrast, isocoumarins have to our knowledge not been characterized as potential MDR-reversing agents to date.

**FIGURE 1:**
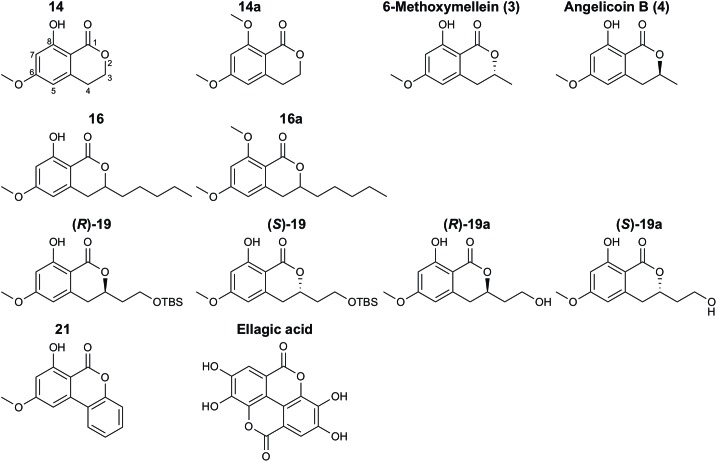
Structures of natural and synthetic 3,4-dihydroisocoumarins.

In this study, the three natural compounds 6-methoxymellein (**3**), angelicoin B (**4**) and ellagic acid as well as nine novel 3,4-dihydroisocoumarins (Figure 1) were analyzed regarding their cytotoxicity in cancer cells and inhibition of the endogenously expressed human ABC transporters P-gp, BCRP, and MRP1 and of the yeast transporter Pdr5. For further insights into the mechanism of action, Pdr5 ATPase and substrate transport assays were performed. These results were complemented with molecular docking studies that indicate that differences in the inhibitory power of the investigated 3,4-dihydroisocoumarins with respect to P-gp-mediated transport result from differences in the compounds’ binding affinities to P-gp.

## Materials and Methods

### Chemicals and Reagents

A549 and HCT-15 cells were purchased from the German Collection of Microorganisms and Cell Cultures (Braunschweig, Germany) and H69AR cells from the American Type Culture Collection (Manassas, VA, United States). Dr. Erasmus Schneider (Wadsworth Center, New York State Department of Health, Albany, NY, United States) kindly provided the MCF-7/MX cells. Cell culture media and supplements were purchased from Thermo Fisher Scientific (Waltham, MA, United States). Yeast extract, peptone, and D-glucose were purchased from Carl Roth (Karlsruhe, Germany). Hoechst 33342, rhodamine 6G (R6G) and adenosine 5′-triphosphate (ATP) were purchased from Sigma-Aldrich (St. Louis, MO, United States) and dissolved in water. Calcein-AM, PSC833, Ko143, MK-571, doxorubicin, mitoxantrone, and ketoconazole (Sigma-Aldrich, St. Louis, MO, United States) were dissolved in dimethyl sulfoxide (DMSO; Carl Roth, Karlsruhe, Germany or Acros Organics, Geel, Belgium). Hepes buffer was purchased from Lonza (Basel, Switzerland). CellTiter-Glo Luminescent Cell Viability Assay was purchased from Promega (Madison, WI, United States). Microtiter plates were purchased from Greiner Bio-One (Kremsmünster, Austria) or Falcon (Corning, NY, United States).

### Test Compounds

3,4-Dihydroisocoumarins were synthesized in three steps starting from α,β-unsaturated δ-lactones (Fischer and Pietruszka, 2007; Böse et al., 2014) and freshly prepared Brassard’s diene. The reaction was catalyzed by AlMe3 as Lewis acid and Tf2CH2 as Brønsted acid leading to the major vinylogous (E)-configured Michael-product and the minor cyclic product. The isolated (E)-configured Michael-product was cyclized with the strong base lithium bis(trimethylsilyl)amide (LHMDS) at -78°C to room temperature for 16 h. Both fractions of the isochromenones from the first and second step were oxidized with 2,3-dichloro-5,6-dicyano-1,4-benzoquinone (DDQ) in toluene at room temperature for 4 h to the desired end product. Overall yields were between 30 and 84%.

Deprotection of isocoumarins **(*R*)-19** and **(*S*)-19** by boron trifluoride diethyl ether in dichloromethane at 0°C gave **(*R*)-19a** and **(*S*)-19a** in good to moderate yields between 36 and 47%. The free hydroxy-groups of the pentyl-derivative **16** and the unsubstituted isocoumarin **14** were protected by dimethylsulfate. This procedure gave 66% of the corresponding methyl-protected pentyl-isocoumarin **16a** and 51% of the methyl-protected isocoumarin **14a**.

**Ellagic acid** was provided by MicroCombiChem GmbH (Wiesbaden, Germany). All test compounds were dissolved in DMSO.

### Cell Culture

#### Human Cancer Cell Lines

The human lung adenocarcinoma cell line A549 was cultured in DMEM medium supplemented with 10% fetal bovine serum (FBS), 100 U/mL penicillin and 100 μg/mL streptomycin. P-gp-expressing human colon adenocarcinoma cells HCT-15 were cultured in RPMI 1640 medium supplemented with 10% FBS, 100 U/mL penicillin and 100 μg/mL streptomycin. BCRP-expressing MCF-7/MX human breast adenocarcinoma cells were cultured in DMEM medium supplemented with 10% FBS, 100 U/mL penicillin and 100 μg/mL streptomycin. MRP1-expressing H69AR human small cell lung cancer cells were cultured in RPMI 1640 medium (ATCC modification) supplemented with 20% FBS, 100 U/mL penicillin and 100 μg/mL streptomycin. All cancer cell lines employed have been systematically characterized regarding their respective ABC transporter expression profile (Sachs et al., 2019).

Cells were maintained in a humidified atmosphere at 37°C and 5% CO_2_ and subcultured when confluency reached 80 to 90%.

#### Yeast Strain

The following *S. cerevisiae* yeast strain was used: YRE1001 (*MATa ura3-52 trp1-1 leu2-3,112 his3-11,15 ade2-1 pdr1-3 pdr5pdr5promΔ::TRP1*). *S. cerevisiae* was cultured in YPD medium (10 g/L yeast extract, 20 g/L peptone, 2% glucose) at 30°C and 200 rpm. The yeast strain has been described previously (Ernst et al., 2008; Gupta et al., 2014). Based on these studies it is evident that Pdr5 localizes to the PM and is present in approximately 10% of the overall membrane protein content of the PM.

### Cell Viability Assay

Cytotoxic activity of test compounds was analyzed after 48 h using the CellTiter-Glo Luminescent Cell Viability Assay published previously (Weber et al., 2017) in 384-well plates with the following cell densities: A549, MCF-7/MX: 2 × 10^3^ cells/well; HCT-15: 3 × 10^3^ cells/well; H69AR: 5 × 10^3^ cells/well. Pipetting was conducted with the CyBi-Well 96-channel simultaneous pipettor (Analytik Jena AG, Jena, Germany). For generation of dose-response curves and calculation of IC_50_ values, the four-parameter logistic model was applied.

The ability of identified inhibitors of P-gp and BCRP to sensitize HCT-15 or MCF-7/MX cells to chemotherapy was analyzed by co-treatment with test compounds and doxorubicin or mitoxantrone, respectively. Cells were incubated with a serial dilution of the chemotherapeutic drug alone or in combination with fixed concentrations of the test compounds or positive controls (2.5 μM PSC833 and 1 μM Ko143, respectively). Fold change of drug IC_50_ was calculated with the formula IC_50_ (drug alone)/IC_50_ (drug with modulator).

### Liquid Drug Assay

The liquid drug assay was carried out in sterile 96-well plates. 50 μl of yeast cell culture at an OD_600_ of 0.15 were mixed with 193.75 μl YPD and 6.25 μl of the test compound or DMSO as a negative control. To distinguish between cytotoxic compounds and those which inhibit Pdr5 WT specifically, the Pdr5 substrate ketoconazole was added only to cultures expressing wild-type Pdr5 in a final concentration of 1 μg/ml. At this concentration, inhibition of Pdr5 would result in ketoconazole-mediated cell death. After 48 h at 30°C the growth was measured with an ELISA plate reader (BioRad, Hercules, CA, United States) at 595 nm.

### Isolation of Pdr5 Containing Plasma Membranes

*Saccharomyces cerevisiae* cells expressing Pdr5 WT or the E1036Q (EQ) mutant were cultured to an OD_600_ of 1.5 in YPD-media at 25°C. The nitrogen source was replenished by addition of a 10^th^ volume of 5x YP (50 g/l yeast extract, 100 g/l peptone). The cells were grown to an OD of 3.5 and harvested (5000 ×*g* at 4°C for 15 min). The isolation of Pdr5 containing PMs was performed as described (Kolaczkowski et al., 1996; Ernst et al., 2008).

### Inhibition of ABC Transporter Activities

#### P-gp Transport Assay

The transport of calcein-AM by P-gp and its modulation by test compounds was analyzed in the P-gp-expressing HCT-15 cell line. 5 × 10^4^ cells were seeded in black 96-well plates and incubated at 37°C and 5% CO_2_ for 24 h. The culture medium was removed and replaced by Hank’s balanced salt solution supplemented with 10 mM HEPES. Test compounds, 2.5 μM PSC833 (positive control) or 0.5% DMSO (negative control) were added in triplicates and incubated for 30 min at 37°C and 5% CO_2_. The substrate calcein-AM was added (final concentration 0.5 μM) and the fluorescence (excitation 485 nm, emission 520 nm) was measured over 3 h with the Infinite m1000 pro microplate reader (Tecan Group AG, Männedorf, Switzerland) at 37°C. The transport activity of P-gp was analyzed by determining the slope of the linear part of the fluorescence-time curve (0–30 min) using linear regression. Normalization of the data to PSC833 (100% inhibition) and DMSO (0% inhibition) was performed.

#### BCRP Transport Assay

Breast cancer resistance protein transport activity was determined in the BCRP-expressing MCF-7/MX cell line. Hoechst 33342 was applied as transporter substrate and 1 μM Ko143 as positive control or 0.5% DMSO as negative control and the assay was performed according to the P-gp assay. Fluorescence was measured at an excitation wavelength of 355 nm and emission wavelength of 460 nm. The transport activity of BCRP was analyzed by determining the plateau of the fluorescence-time curve by using non-linear regression (one-phase exponential fit). Normalization of the data to Ko143 (100% inhibition) and DMSO (0% inhibition) was performed.

#### MRP1 Transport Assay

Transport activity of MRP1 was determined in MPR1-expressing H69AR cells using the substrate calcein-AM and 20 μM MK-571 as the positive control or 0.5% DMSO as negative control. Experimental procedure was according to the P-gp assay, except that 7.5 × 10^4^ cells were seeded per well. The transport activity of MRP1 was analyzed by determining the slope of the linear part of the fluorescence-time curve (0–20 min) using linear regression. Normalization of the data to MK-571 (100% inhibition) and DMSO (0% inhibition) was performed.

#### Pdr5 Transport Assay

Active transport of R6G was measured according to the protocol developed by Kolaczkowski et al. (1996), using a Tecan Infinite 200 pro reader (Tecan Group AG, Männedorf, Switzerland). 6 μg of the isolated PM containing Pdr5 were resuspended in 200 μl of the transport buffer (50 mM HEPES, pH 7.0, 5 mM MgCl_2_, 10 mM NaN_3_, and 150 nM R6G) and incubated in a black 96-well plate at 30°C. 5 μl of the test compound were added at the indicated concentrations. Active transport was initiated by the addition of 10 μl 200 mM ATP and fluorescence intensity was recorded in 15 s-intervals for 20 min (excitation wavelength at 524 nm, emission wavelength at 558 nm, number of flashes 30, integration time 2000 μs). IC_50_ determination was performed with a serial dilution of the test compounds.

### Pdr5 ATPase Assay

Oligomycin (OM)-sensitive ATPase activity was measured from 0.8 μg PM containing Pdr5 incubated with 2 mM ATP, 5 mM MgCl_2_ in 270 mM TRIS-glycine buffer (pH 9.5) and 2.5 μl of the test compounds or DMSO as a control in a total volume of 100 μl. To reduce the background activities, 0.2 mM ammonium molybdate, 10 mM NaN_3_ and 50 mM KNO_3_, respectively, were added (Dufour et al., 1988; Goffeau and Dufour, 1988). In a second assay, OM (20 μg/ml) was added to an otherwise identical setup. After incubation at 30°C for 20 min, the reaction was stopped by adding 25 μl of the reaction to 175 μl 40 mM H_2_SO_4_. The amount of released inorganic phosphate was determined by a colometric assay in 96-well plates (Goffeau and Dufour, 1988; Decottignies et al., 1994; Wada et al., 2002). The difference of both assays ( ± OM) corresponds to the specific ATPase activity of Pdr5 (Ernst et al., 2008).

### Molecular Docking Studies

The cryo-EM structure of P-gp (PDB ID: 6FN1, chains A and B) (Alam et al., 2018) was prepared for molecular docking using the Protein Preparation Wizard (Sastry et al., 2013) as implemented in the Maestro GUI^1^ of the Schrödinger Suite version 2018-2^1^. Protonation states for Asp, Glu, His and Lys, tautomers for His and chi flips for Asp, Glu, and His were calculated at pH 7.4 with the PROPKA (Olsson et al., 2011; Søndergaard et al., 2011) implementation in Maestro. Subsequently, a restrained energy minimization was performed on all hydrogen atoms. To be able to use the structure for later MM-GBSA calculations with implicit membrane, the coordinates of the prepared structure were superimposed onto the coordinates of its respective OPM (Lomize et al., 2012) entry. The membrane region was then defined in the Maestro GUI, using the membrane dimensions of 31.4 Å calculated by the PPM server (Lomize et al., 2012).

Molecular docking was performed using the Glide XP docking protocol (Friesner et al., 2004, 2006) of the Schrödinger Suite version 2018-2. To allow more poses to pass through the initial Glide screens, the initial number of poses per ligand was increased from 5,000 to 50,000 and the scoring window was widened from 100.0 to 500.0. In addition, the number of minimized poses per ligand was increased from 400 to 1,000 and the ‘expanded sampling’ option was enabled. Post-docking minimization was performed on 100 poses, and the resulting best 10 poses per ligand were kept for further evaluation.

Four re-docking experiments were performed to assess the suitability of the selected docking approach: two re-dockings were performed in the presence of the respective other ligand entity (re-docking 1), and two re-dockings were performed in which both ligand entities were docked sequentially (re-docking 2). For re-docking 1, the size of the region in which the geometric center of the ligand can move during docking (‘inner box’) was set to 10 Å, and the size of the region in which ligand atoms may be placed during docking (‘outer box’) was set such that it expands the size of the inner box by 17 Å. For re-docking 2, the center and size of the inner box was set to match the center and size of the smallest possible cuboid that encloses both ligands in the cryo-EM complex. The size of the outer box was set such that it expands the size of the inner box by 15 Å. The same box as for re-docking 2 was also used for the docking of the 3,4-dihydroisocoumarins. Since this docking procedure produced favorably scored poses in two different binding sites, the dockings were repeated for each of these binding sites individually, using docking boxes centered on the geometric center of the poses generated in the corresponding binding pocket. For these dockings, the box sizes were set as for re-docking 1.

In order to obtain a more reliable estimate of the relative affinities of the 3,4-dihydroisocoumarins, for each of the two binding sites, the best scored pose of each docked ligand was post-processed using the MM-GBSA implementation in Prime (see footnote number 1) with the VSGB 2.1 solvation model (Jianing et al., 2011). During this procedure, protein flexibility was taken into account for all residues within 8 Å of the ligand pose. Contributions due to changes in the configurational entropy of the ligand or the receptor upon complex formation were neglected, in order to avoid introducing uncertainty in the computations (Gohlke and Case, 2004; Weis et al., 2006; Hou et al., 2011). For compounds **16** and **16a**, the final effective binding energy was expressed as the average over the effective binding energies for both enantiomers. All effective binding energies were then converted to relative effective binding energies.

### Data Analysis and Statistics

All experiments were performed in at least three independent replicates. Data were analyzed with GraphPad Prism v. 6.07 (GraphPad Software, Inc., La Jolla, CA, United States) and results are presented as mean ± SEM. Different groups were compared statistically using one-way ANOVA followed by Dunnett’s multiple comparisons test. Differences were considered significant when *p* < 0.05.

## Results

### Cytotoxic Activity of 3,4-Dihydroisocoumarins in Human Cancer Cell Lines

Cytotoxicity of 12 3,4-dihydroisocoumarins was analyzed in the sensitive lung cancer cell line A549 as well as in three resistant cell lines HCT-15 (colon carcinoma), MCF-7/MX (breast carcinoma) and H69AR (lung carcinoma) either expressing the ABC transporter P-gp, BCRP, or MRP1, respectively. Cells were incubated for 48 h with test compounds and cell viability was assessed.

IC_50_ values of the tested 3,4-dihydroisocoumarins are summarized in Table 1. Most compounds did not display significant cytotoxic activities in the cancer cell lines and IC_50_ values were above the highest concentrations applied in the assay. However, one exception were the enantiomers **(*R*)-19** and **(*S*)-19**. Both compounds displayed comparable cytotoxicity in sensitive A549 cells and the BCRP-overexpressing MCF-7/MX cells. In A549 cells, IC_50_ value of compound **(*R*)-19** was 47.5 ± 3.2 μM (mean ± SEM) and IC_50_ value of **(*S*)-19** was 42.0 ± 3.9 μM. In MCF-7/MX cells, both compounds were slightly more toxic with IC_50_ values of 28.1 ± 0.7 μM and 33.5 ± 7.5 μM, respectively. In contrast, in P-gp expressing HCT-15 cells, only **(*R*)-19** demonstrated a cytotoxic effect, whereas **(*S*)-19** was not toxic. Furthermore, the unprotected derivatives of **(*R*)-19** and **(*S*)-19**, **(*R*)-19a** and **(*S*)-19a**, were analyzed. In both cases, removal of the protecting TBS group led to complete or near complete loss of cytotoxic activity in all cell lines.

**Table 1:** Cytotoxicity of 3,4-dihydroisocoumarins on human tumor cell lines.

Compound	IC_50_ (μM)			
	A549	HCT-15	MCF-7/MX	H69AR
14	>100	>100	>100	>100
14a	>100	>100	>100	>100
16	78.6 ± 2.2	>100	>100	88.5 ± 4.6
16a	53.6 ± 5.6	>100	10.6 ± 0.9	>100
(*R*)-19	47.5 ± 3.2	57.1 ± 7.5	28.1 ± 0.7	>100
(*R)*-19a	>100	>100	>100	>100
(*S*)-19	42.0 ± 3.9	>100	33.5 ± 7.5	86.8 ± 9.2
(*S*)-19a	>100	>100	89.8 ± 4.9	>100
6-Methoxymellein (3)	>100	>100	>100	>100
Angelicoin B (4)	>100	>100	>100	>100
21	>50	>50	>50	>50
Ellagic acid	>50	>50	>50	>50


*Data represent mean ± SEM of at least three independent experiments performed in quadruplicates.*

Compound **16** was not cytotoxic in P-gp expressing HCT-15 cells and BCRP-expressing MCF-7/MX cells. In MRP1-expressing H69AR cells and sensitive A549 cells, only minor cytotoxic activity was observed. IC_50_ values were 88.5 ± 4.6 μM and 78.6 ± 2.2 μM, respectively. Remarkably, the derivative **16a**, which differs from **16** by the methoxy group instead of the hydroxy group at position 8, displayed slightly improved activity in A549 cells with an about 1.5-fold lower IC_50_ value and a clearly improved activity in BCRP-expressing MCF-7/MX cells. In those cells, the IC_50_ value was 10.6 ± 0.9 μM. In P-gp or MRP1-expressing cell lines, no cytotoxicity could be observed.

The known natural products 6-methoxymellein (**3**), angelicoin B (**4**) and **ellagic acid** did not show any cytotoxic effects against the tested cancer cell lines.

### Identification of 3,4-Dihydroisocoumarins as Dual Inhibitors of P-gp and BCRP

**FIGURE 2:**
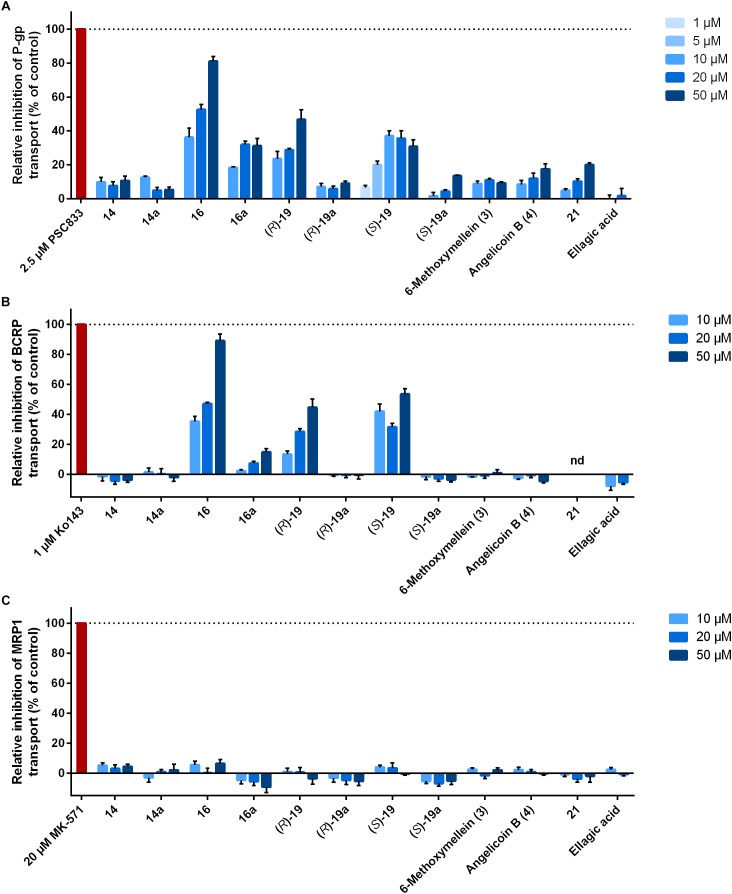
Influence of 3,4-dihydroisocoumarins on ABC transporter activity. Cancer cell lines overexpressing **(A)** P-gp (HCT-15), **(B)** BCRP (MCF-7/MX), or **(C)** MRP1 (H69AR) were incubated with test compounds and intracellular fluorescence of the substrates calcein-AM (P-gp, MRP1) or Hoechst 33342 (BCRP) was measured over 3 h. Data were normalized to DMSO (0% inhibition) and the positive controls PSC833, Ko143, and MK-571, respectively (100% inhibition). Bars represent mean ± SEM of at least three independent experiments. nd, not determined because of intrinsic fluorescence.

The 12 3,4-dihydroisocoumarins were tested for their ability to inhibit the transport activity of the human ABC transporters P-gp, BCRP, and MRP1 in cell lines overexpressing the respective transporter. Therefore, intracellular fluorescence of the transporter substrates calcein-AM and Hoechst 33341 were measured over time after cells had been treated with different concentrations of the test compounds. Inhibition rates of the compounds were normalized to the positive controls 2.5 μM PSC833, 1 μM Ko143, and 20 μM MK-571, respectively. As a result, three compounds were identified as dual inhibitors of transport activity of P-gp and BCRP (Figure 2A,B).

Pentyl-derivative **16** was the strongest dose-dependent inhibitor of P-gp and BCRP among the tested compounds. Relative inhibition of P-gp was 36.3 ± 5.3% at a concentration of 10 μM, 52.6 ± 3.1% at 20 μM and 81.2 ± 2.7% at 50 μM (mean ± SEM). Regarding inhibition of BCRP, the rates were comparable to P-gp with relative values between 35.3 ± 3.4% at 10 μM and 89.1 ± 4.6% at 50 μM. Interestingly, derivative **16a**, which differs from **16** only by the methoxy group instead of the hydroxy group at position 8, showed reduced inhibition rates against both transporters with a more pronounced effect for BCRP. Relative inhibition was between 18.4 ± 0.5 and 31.9 ± 2.2% for P-gp and 2.3 ± 0.9 and 14.9 ± 2.2% for BCRP.

Two additional compounds were identified as dual P-gp and BCRP inhibitors, **(*R*)-19** and **(*S*)-19**, but with lower activities compared to derivative **16**. Derivative **(*R*)-19** inhibited P-gp with relative rates between 23.6 ± 4.2 and 46.8 ± 5.7%. Regarding **(*S*)-19**, highest P-gp inhibition was achieved at 10 μM (37.1 ± 2.9%). Inhibition of BCRP was comparable. As both compounds contain protective groups at the hydroxy group at the ethyl chain at position 3, inhibition rates were compared to the deprotected derivatives **(*R*)-19a** and **(*S*)-19a**. In both cases, the deprotected compounds showed clearly reduced inhibitory ability of P-gp with values between about 5 and 15% and completely abolished inhibitory ability of BCRP.

Two compounds without substitution at position 3 were analyzed, **14** and **14a**. They only differ in the substituent at position 8, which is either a hydroxy group **14** or methoxy group **14a**. Both compounds did not inhibit BCRP and displayed only minor inhibitory activity against P-gp up to about 10% relative inhibition.

Furthermore, the two natural products and enantiomers 6-methoxymellein (**3)** and angelicoin B (**4)** were studied. They share the same structure with compound **14**, but are substituted with a methyl group at position 3. Relative rates of P-gp inhibition of 6-methoxymellein (**3)** and angelicoin B (**4)** did not differ significantly from those of derivative **14**. In case of BCRP, no inhibitory activity could be observed, as was the case for derivative **14**.

Compound **21**, a tricyclic derivative, weakly inhibited P-gp with a relative inhibition rate of 20.0 ± 1.2% at the highest concentration used. Its ability to inhibit BCRP could not be determined due to inherent fluorescence. The natural product **ellagic acid** did not display any inhibitory activity against P-gp or BCRP. It has to be noted that this compound could not be employed at a concentration of 50 μM due to its low solubility.

Regarding inhibition of MRP1 transport function in H69AR cells, none of the test compounds demonstrated any activity (Figure 2C).

### Sensitization of Resistant Cancer Cells to Chemotherapy by 3,4-Dihydroisocoumarins

Cancer cells overexpressing MDR related ABC transporters are characterized by reduced susceptibility to chemotherapeutic drugs, which are substrates of the respective transporters. These are for example doxorubicin (P-gp) and mitoxantrone (BCRP). To determine if the identified P-gp and BCRP inhibitor derivative **16** is able to re-sensitize resistant cancer cells to chemotherapy, P-gp expressing HCT-15 cells and BCRP-expressing MCF-7/MX cells were co-treated with doxorubicin or mitoxantrone and different concentrations of compound **16**. IC_50_ values were determined after 48 h and compared to single treatment with the chemotherapeutic drugs. PSC833 (2.5 μM) and Ko143 (1 μM) served as respective positive controls for P-gp and BCRP, respectively.

**FIGURE 3:**
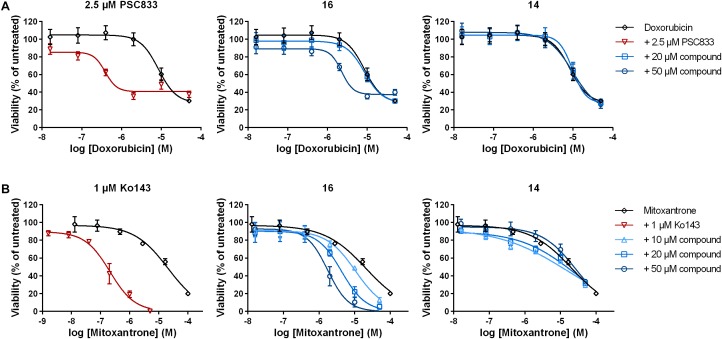
Sensitization of resistant cancer cells to chemotherapy by selected 3,4-dihydroisocoumarins. **(A)** P-gp expressing HCT-15 and **(B)** BCRP-expressing MCF-7/MX were treated with either doxorubicin or mitoxantrone alone, in combination with positive controls PSC833 or Ko143 or in combination with varying concentrations of test compounds **16** and **14**. Cell viability was determined after 48 h. Data represent mean ± SEM normalized to untreated cells of at least three independent experiments performed in quadruplicates.

In both cases, compound **16** was able to significantly sensitize the resistant cancer cells to chemotherapy. In HCT-15 cells, doxorubicin alone had an IC_50_ value of 8.5 ± 0.7 μM (mean ± SEM; Figure 3A and Table 2). Addition of 20 μM of compound **16** did not lead to a change in IC_50_, whereas the combination with 50 μM of the compound lead to a 3.7-fold decrease in IC_50_ to 2.3 ± 0.1 μM. In contrast, compound **14**, which displayed minor P-gp inhibiting activity, did not sensitize the cells to doxorubicin. The positive control PSC833 reduced the IC_50_ of doxorubicin around 28-fold to a value of 0.35 ± 0.08 μM.

**Table 2:** Sensitization of HCT-15 cells to doxorubicin treatment by selected 3,4-dihydroisocoumarins.

Compound	IC_50_ (μM)	*p*-Value	Fold change
Doxorubicin	8.5 ± 0.7	–	1.00
			
+2.5 μM PSC833	0.3 ± 0.03^∗∗^	0.0017	28.33
			
+20 μM 16	8.7 ± 1.6	0.9999	0.98
+50 μM 16	2.3 ± 0.08^∗^	0.0129	3.70
			
+20 μM 14	9.6 ± 1.0	0.9498	0.89
+50 μM 14	10.6 ± 2.1	0.6313	0.80


*PSC833 was used as positive control. Fold change of cytotoxicity was determined by dividing the IC_*50*_ value of cells treated with doxorubicin alone by the IC_*50*_ value of cells treated with doxorubicin in combination with the respective test compound. Data show mean ± SEM of three independent experiments performed in quadruplicates. ^∗^*p* < 0.05; ^∗∗^*p* < 0.01 compared to doxorubicin alone (one-way ANOVA followed by Dunnett’s multiple comparisons test).*

In BCRP-expressing MCF-7/MX cells, IC_50_ value of the drug mitoxantrone alone was 19.6 ± 2.4 μM (Figure 3B and Table 3). Addition of compound **16** revealed a dose-dependent decrease in IC_50_ up to 5.6-fold at 50 μM to 3.5 ± 0.9 μM. As observed above, compound **14**, which did not inhibit BCRP transport function, did not have a significant influence on the sensitivity of the cells to mitoxantrone. The positive control Ko143 was able to increase the cytotoxicity of mitoxantrone around 65-fold to an IC_50_ value of 0.33 ± 0.07 μM.

**Table 3:** Sensitization of MCF-7/MX cells to mitoxantrone treatment by selected 3,4-dihydroisocoumarins.

Compound	IC_50_ (μM)	*p*-Value	Fold change
Mitoxantrone	19.6 ± 2.4	–	1.00
+1 μM Ko143	0.3 ± 0.07^∗∗∗∗^	< 0.0001	65.33
+10 μM 16	21.1 ± 1.4	0.9795	0.93
+20 μM 16	5.3 ± 1.1^∗∗∗∗^	< 0.0001	3.70
+50 μM 16	3.5 ± 0.9^∗∗∗∗^	< 0.0001	5.60
+10 μM 14	19.4 ± 2.6	> 0.9999	1.01
+20 μM 14	25.5 ± 1.0	0.0942	0.77
+50 μM 14	25.0 ± 1.3	0.0988	0.78


*Ko143 was used as positive control. Fold change of cytotoxicity was determined by dividing the IC_*50*_ value of cells treated with mitoxantrone alone by the IC_*50*_ value of cells treated with mitoxantrone in combination with the respective test compound. Data show mean ± SEM of three independent experiments performed in quadruplicates. ^∗∗∗∗^*p* < 0.0001 compared to doxorubicin alone (one-way ANOVA followed by Dunnett’s multiple comparisons test).*

### Cytotocity of 3,4-Dihydroisocoumarins on Pdr5 Wild-Type and Pdr5 EQ Expressing Yeast Strains and Their Ability to Inhibit Transport and ATPase Activity of Pdr5 Wild-Type

**FIGURE 4:**
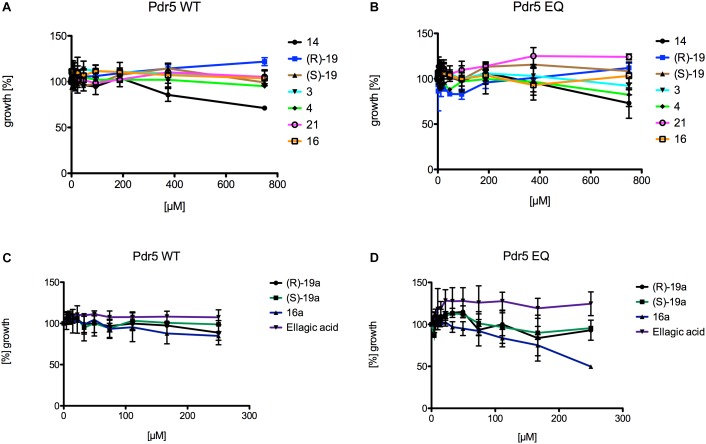
Liquid drug assays of the 12 3,4-dihydroisocoumarins and *S. cerevisiae* strains expressing Pdr5 WT or the mutant Pdr5 EQ. **(A)** The influence of **14**, ***(R)*-19**, ***(S)*-19**, **3**, **4**, **21**, and **16** on growth of strains expressing Pdr5 WT. **(B)** The influence of **14**, ***(R)*-19**, ***(S)*-19**, **3**, **4**, **21**, and **16** on growth of strains expressing Pdr5 EQ. **(C)** The influence of ***(R)*-19a**, ***(S)*-19a**, **16a** and **ellagic acid** on growth of strains expressing Pdr5 WT. **(D)** The influence of ***(R)*-19a**, ***(S)*-19a**, **16a** and **ellagic acid** on growth of strains expressing Pdr5 EQ. Cells were incubated at different concentrations of the compounds for 48 h at 30 °C and OD_600_ was determined.

The 12 3,4-dihydroisocoumarins were tested on two different *S. cerevisiae* strains to determine their cytotoxicity on yeast. Therefore, a Pdr5 WT and a Pdr5 EQ mutant overexpressing strain were incubated with serial dilutions of the test compounds and OD_600_ was measured after 48 h of cell growth at 30°C. None of the tested 3,4-dihydroisocoumarins showed an effect on the growth of the cells (Figure 4), except compound **16a**, which inhibited the growth of the mutant strain slightly at the highest tested concentration (Figure 4D). However, it had no effect on the wild-type strain (Figure 4C).

**FIGURE 5:**
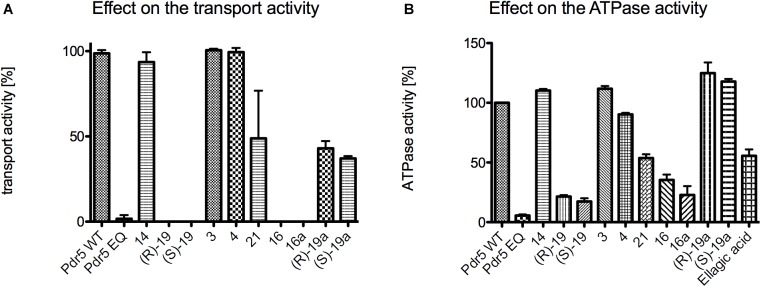
Initial screening of the 12 test compounds to determine their effect on **(A)** the transport activity of Pdr5 WT and **(B)** their effect on the ATPase activity. Within the transport assay compound **14**, **3** and **4** had no effect on both activities. Compound **21**, ***(R)*-19a** and ***(S)*-19a** were able to inhibit the transport activity to around 50%. Only compound ***(R)*-19**, ***(S)*-19**, **16** and **16a** were able to inhibit the transport activity of Pdr5 WT completely. In contrast, compounds **14**, **3**, **4**, ***(R)*-19a** and ***(S)*-19a** had nearly no effect on the ATPase activity. Compound **21** and **ellagic acid** were able to inhibit the ATPase activity of Pdr5 WT to 50%. Only compound ***(R)*-19**, ***(S)*-19**, **16** and **16a** were able to inhibit ATPase activity below 40% in comparison to Pdr5 WT in the absence of any compound.

In a first initial screen, the effect of the highest concentrations (10 mM) of the 3,4-dihydroisocoumarins on R6G transport and ATPase activity of Pdr5 in PM preparations was analyzed (Figure 5). In the case of the transport assay, Pdr5 containing PM preparations were incubated simultaneously with R6G and the test compound. If Pdr5 actively transports R6G, the concentration of the substrate increases in one of the membrane leaflets, which leads to a self-quenching effect. This can be measured as a decrease of fluorescence intensity in real time. Compound **14**, 6-methoxymellein (**3)** and angelicoin B (**4)** did not interfere with Pdr5-mediated R6G transport (Figure 5A) as the observed changes in fluorescence were comparable to R6G transport of the wild-type protein in the absence of the compound. In contrast, compounds **21**, ***(R)*-19a** and ***(S)*-19a** inhibited transport activity to 49, 43, and 37%, respectively. Only compound ***(R)*-19**, ***(S)*-19**, **16** and **16a** were able to inhibit the transport of R6G completely.

Figure 5B summarizes the effect of the 3,4-dihydroisocoumarins on ATPase activity of Pdr5 WT. Again, compound **14**, 6-methoxymellein (**3)** and angelicoin B (**4)** had no inhibitory effect. In contrast to the capability of **(R)-19a** and **(S)-19a** to inhibit transport of R6G, no inhibition of the ATPase activity was observed. **Ellagic acid** and compound **21** inhibited the ATPase activity to 56 and 54%, respectively. Compounds ***(R)*-19**, ***(S)*-19**, **16,** and **16a** showed the highest inhibition potential on the Pdr5 WT ATPase activity.

**FIGURE 6:**
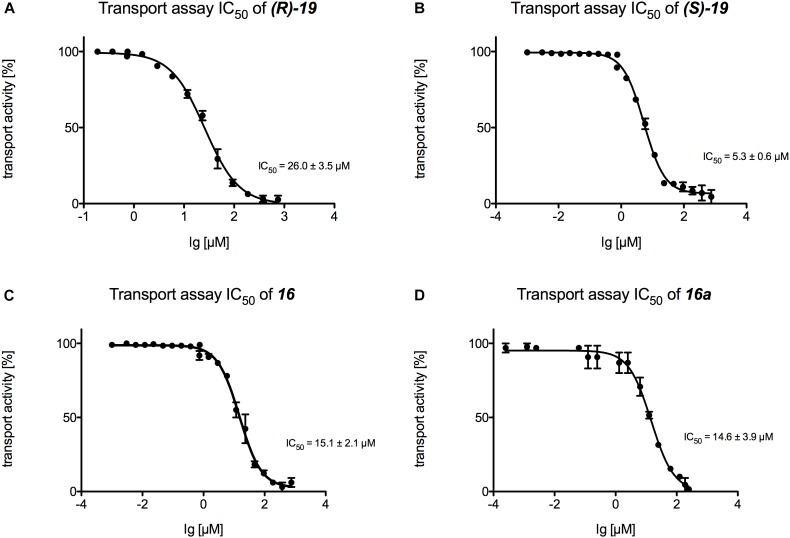
Determination of the transport IC_50_ values for **(A)** compound ***(R)-*19**, **(B)** compound ***(S)-*19**, **(C)** compound **16** and **(D)** compound **16a**. For each compound a serial dilution was used to determine the IC_50_ curve. Membranes were incubated with the compound and active transport was detected with the Tecan Infinite M200.

**FIGURE 7:**
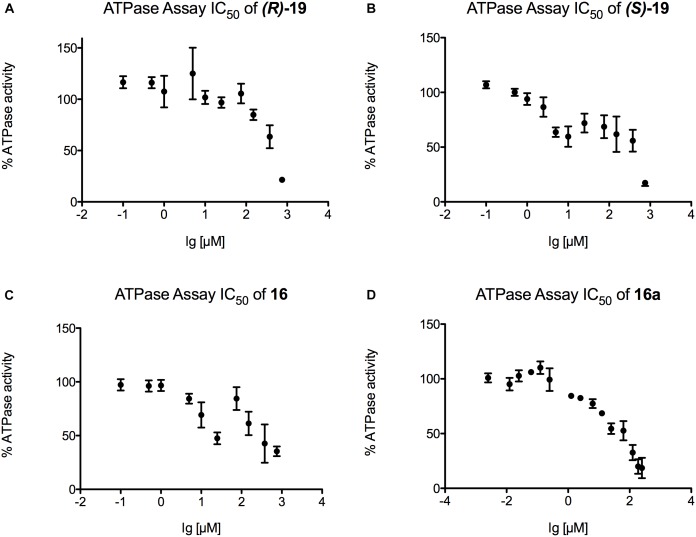
Determination of the ATPase IC_50_ values for **(A)** compound ***(R)-*19**, **(B)** compound ***(S)-*19**, **(C)** compound **16** and **(D)** compound **16a**. For each compound a serial dilution was used to display the IC_50_ curve. Pdr5 plasma membranes (PMs) were incubated with the compound for 20 min and release of inorganic phosphate was detected with an Elisa PlateReader at 595 nm.

Finally, IC_50_ values were determined for those inhibitors that displayed the highest potential in the transport and the ATPase assay (***(R)*-19**, ***(S)*-19**, **16,** and **16a**) (Figure 6, 7). Figure 6 summarizes the IC_50_ measurements of the transport assays. The lowest IC_50_ was determined for compound ***(S)*-19** with 5.3 ± 0.6 μM. Compound **16** and **16a** showed IC_50_ values of 15.1 ± 2.1 and 14.6 ± 3.9 μM, respectively. The highest IC_50_ value was detected for compound ***(R)*-19** with 26.0 ± 3.5 μM. Figure 7 summarizes the IC_50_ measurements with respect to inhibition of ATPase activity. Due to the solubility limit of these compounds, no reliable determination of IC_50_ values could be performed.

### Binding Mode Prediction and Effective Binding Energy Calculations on Complexes of P-Glycoprotein and 3,4-Dihydroisocoumarins

**FIGURE 8:**
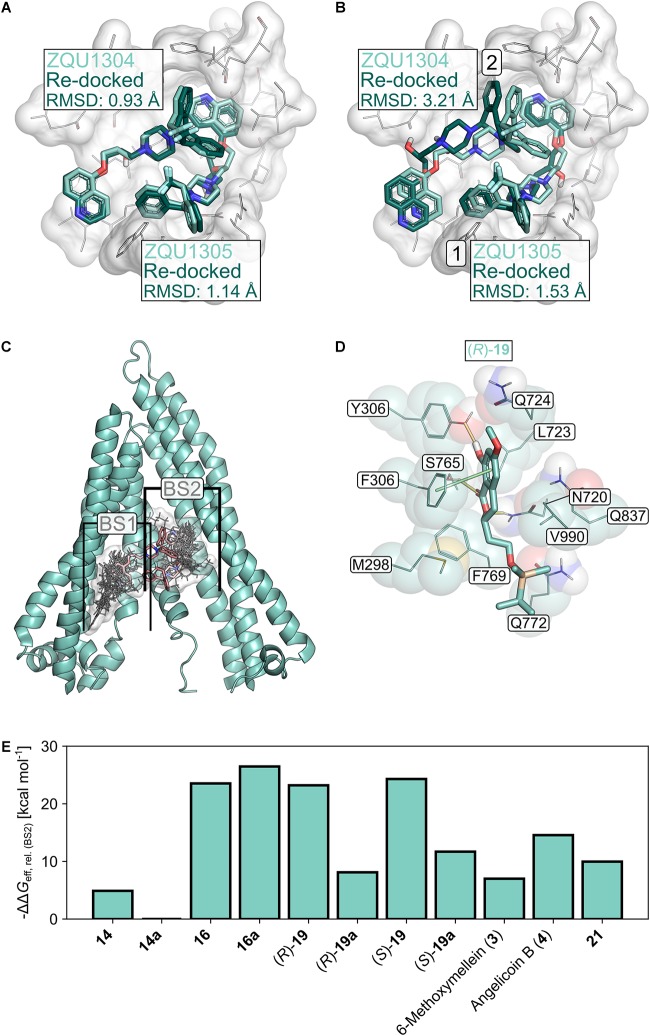
Molecular docking studies. **(A)** Re-docking of Zosuquidar (ZQU, darker turquoise, sticks representation) into the cryo-EM structure (PDB ID: 6FN1; Alam et al., 2018) of P-gp (white, sticks and cartoon representation, only part of the protein is shown for clarity) while the coordinates of the respective second ZQU molecule (lighter turquoise) were retained from the cryo-EM structure. **(B)** Sequential re-docking of ZQU into the cryo-EM structure of P-gp. The result of the first and second runs are indicated with a *1* and *2*, respectively. Colors and representations are as in **(A)**. **(C)** Binding sites (denoted as BS1 and BS2) for 3,4-dihydroisocoumarins found during docking. The molecular structures of the 3,4-dihydroisocoumarins are displayed as lines, and their joint molecular surface is rendered in white. Zosuquidar is shown as red sticks. The relevant region in the transmembrane domain of P-gp is shown in cartoon representation **(D)** Binding mode of **(*R*)-19** in BS2 of P-gp. Hydrogen bonds are displayed as yellow lines, π-stacking interactions as green lines. **(E)** Relative effective binding energies for 3,4-dihydroisocoumarins in P-gp, calculated by the MM-GBSA approach. To allow for a better comparison with the P-gp transport assay, the energies are expressed as -ΔΔ*G*_eff_.

The selected docking protocol was able to reproduce the binding modes of Zosuquidar (ZQU) in P-gp, regardless of whether the second ligand entity was retained in the structure (RMSD_ZQU1304_: 0.93 Å, RMSD_ZQU1305_: 1.14 Å, Figure 8A) or not (RMSD_ZQU1305_: 1.53 Å, RMSD_ZQU1304_: 3.21 Å, Figure 8B), suggesting that it is also suitable for predicting the binding modes of other molecules, such as the 3,4-dihydroisocoumarins. When two molecules of ZQU were sequentially docked to the P-gp structure, all poses obtained during the first run corresponded to the binding mode of ZQU1305 in the cryo-EM structure (Figure 8B), indicating that the subpocket occupied by this ligand entity has a higher affinity toward ZQU than the subpocket occupied by the ligand entity ZQU1304. In line with this, the pose obtained for re-docking of ZQU1305 with ZQU1304 present was scored markedly better than the pose obtained for re-docking of ZQU1304 with ZQU1305 present (Glide Docking Scores: -10.40 and -8.60 kcal mol^-1^, respectively). Similarly, initial docking of the 3,4-dihydroisocoumarins revealed two spatially distinct binding sites (BS1 and BS2) overlapping with those of ZQU1304 and ZQU1305, respectively, to which all ligands were docked separately during the final docking experiment (Figure 8C). Since, on average, the calculated effective binding energies were 7.44 ± 3.45 kcal mol^-1^ (21.64 ± 6.01 kcal mol^-1^ for the active molecules **16**, **(*R*)-19** and **(*S*)**-**19**) more favorable in BS2 (Table 4), only the poses in BS2 were used for the final evaluation.

**Table 4:** Relative effective energies of 3,4-dihydroisocoumarins binding to P-gp.^1^

Compound	ΔΔ*G*_eff_^2^	
	BS1	BS2
14	–10.04 (–24.55)	–14.16 (–28.67)
14a	–14.85 (–29.36)	–9.27 (–23.78)
16	–13.46 (–27.97)	–32.80 (–47.31)
16a	–24.12 (–38.63)	–35.74 (–50.25)
(*R*)-19	–20.48 (–34.99)	–32.48 (–46.99)
(*R)*-19a	–22.61 (–37.12)	–17.38 (–31.89)
(*S*)-19	0.00 (–14.51)	–33.57 (–48.08)
(*S*)-19a	–14.41 (–28.92)	–20.96 (–35.47)
6-Methoxymellein (3)	–12.62 (–27.13)	–16.27 (–30.78)
Angelicoin B (4)	–20.66 (–35.17)	–23.83 (–38.34)
21	–20.56 (–35.02)	–19.23 (–33.74)


*^*1*^All values are relative to the effective binding energy of (*S*)-19 in BS1. ^*2*^In kcal mol^-*1*^.*

The calculated effective binding energies of the 3,4-dihydroisocoumarins agree very well with their ability to inhibit P-gp-mediated transport: Compounds **16**, **16a**, **(*R*)-19** and **(*S*)-19**, which all inhibited substrate transport by > 20% at concentrations of 20 μM (Figure 2A), also showed significantly more favorable effective binding energies than the inactive compounds (Δ*G*_eff_ = -48.16 ± 0.73 kcal mol^-1^ versus Δ*G*_eff_ = -31.81 ± 1.79 kcal mol^-1^, *p* < 10^-4^) (Figure 8C and Table 4). Note, though, that configurational entropy differences were neglected here, such that the difference in the binding free energies of the two classes may be smaller due to enthalpy-entropy compensation effects. These results validate the obtained binding modes for the 3,4-dihydroisocoumarins in P-gp and corroborate the experimental findings on the compounds’ ability to inhibit P-gp-mediated substrate transport.

## Discussion

In this study, 3,4-dihydroisocoumarins, which have so far not been in the focus as potential MDR reversing agents, were evaluated as potential inhibitors of human and yeast ABC transporters. Notably, among the tested derivatives, three novel 3,4-dihydroisocoumarins were identified as dual inhibitors of two human transporters, P-gp and BCRP. Furthermore, the most potent inhibitor, derivative **16**, demonstrated inhibition of both transporters between 80 and 90% compared to the respective positive controls at the highest test concentration (50 μM). As proof of concept, P-gp expressing HCT-15 cells and BCRP-expressing MCF-7/MX cells (Sachs et al., 2019) were treated with compound **16** in combination with the chemotherapeutic drugs doxorubicin and mitoxantrone, broadly described as transporter substrates for P-gp and BCRP. Subsequently, co-treatment with 50 μM of derivative **16** sensitized chemotherapy-resistant cancer cells HCT-15 colon carcinoma cells to doxorubicin by decreasing the IC_50_ value of doxorubicin-induced cytotoxicity by 3.7-fold and sensitized MCF-7/MX breast carcinoma cells to mitoxantrone by decreasing the IC_50_ value of mitoxantrone-induced cytotoxicity by 5.6-fold. Moreover, enantiomers **(*R*)-19** and **(*S*)-19** inhibited transport function of P-gp and BCRP, but with lower potency compared to derivative **16**. Noteworthy, distinct structure-activity relationships for transporter inhibition could be identified, and they run in parallel for P-gp and BCRP: our data show that the hydroxy group at position 6 is mandatory for transporter inhibition and substitution by a methoxy group clearly reduces activity. Furthermore, a hydrophobic carbon chain at position 3 is indispensable for inhibition and this substituent seems to need a certain chain length. Compounds without alkyl substituent (derivative **14**) or with a methyl group [6-methoxymellein (**3)** and angelicoin B (**4)**] as well as compounds with a hydrophilic chain (derivatives **(*R*)-19a** and **(*S*)-19a**) demonstrated significantly decreased inhibition of both transporters. Those effects were more pronounced in BCRP inhibition than in P-gp inhibition.

**FIGURE 9:**
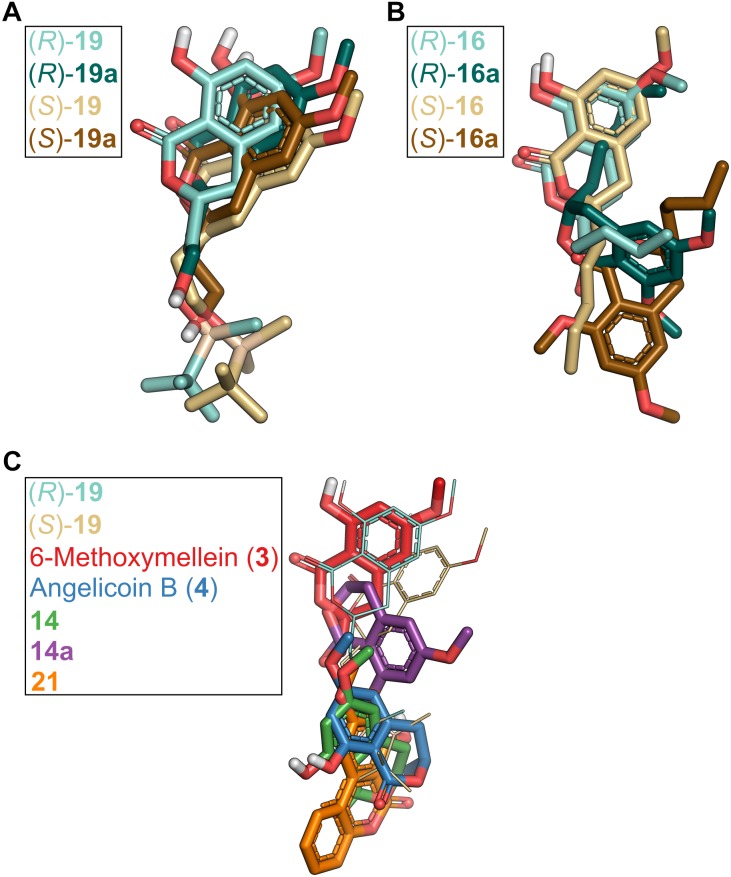
Comparison of binding poses of 3,4-dihydroisocoumarins in P-gp obtained by molecular docking. **(A)** Binding poses of compounds carrying a TBS group in the side chain [**(*R*)-19**, **(*S*)-19**] versus compounds in which the TBS group was removed [**(*R*)-19a**, **(*S*)-19a**]. **(B)** Binding poses of compounds that carry a hydroxy group at position 8 [**(*R*)-16**, **(*S*)-16**] versus compounds that carry a methoxy group at position 8 [**(*R*)-16a**, **(*S*)-16a**]. **(C)** Binding modes of compounds **3**, **4**, **14**, **14a,** and **21** in comparison to **(*R*)-19** and **(*S*)-19**.

Molecular docking can provide an explanation at the atomistic level for the observed structure-activity relationship. Here, we focused on docking of 3,4-dihydroisocoumarins to P-gp because for this system it was possible to validate the suitability of our docking protocol. The predicted binding modes of the active molecules **16**, **(*R*)-19** and **(*S*)-19** close to the binding sites of ZQU are largely identical (Figure 8D,E, 9A,B) and reveal that the hydroxy group at position 8 stabilizes the binding mode via hydrogen bonding to the hydroxy group in Y306. Introduction of a bulky methyl group, as realized in **16a**, not only abolishes this interaction, but also leads to a steric clash with Y306, forcing the molecule to invert its orientation (Figure 9B). Furthermore, the hydrophobic OTBS moiety in **(*R*)-19** and **(*S*)-19** protrudes into a predominantly hydrophobic subpocket, formed by residues M298, N720, F769, Q772, Q837, and V990. Removal of this group, as realized in **(*R*)-19a** and **(*S*)-19a**, leads to an unsatisfied hydrogen bond donor in this subpocket, which disfavors binding. Finally, compounds **3**, **4**, **14**, **14a**, and **21** adopt binding modes that either resemble the inverted orientation of **16a**, or are dissimilar to those of the active molecules **16**, **(*R*)-19** and **(*S*)-19** (Figure 9C). Taken together, these results indicate that differences in the inhibitory power of the investigated 3,4-dihydroisocoumarins with respect to P-gp-mediated transport result from differences in the compounds’ binding affinities to P-gp rather than their ability to modulate P-gp’s transport cycle or to affect P-gp function via a different mechanism. They further support the recent finding (Alam et al., 2018) that the central cavity in P-gp can accommodate multiple molecules in different subpockets and, thereby, substantiate the concept of a binding site with high plasticity (Wise, 2012; McCormick et al., 2015).

Regarding these results, the 3,4-dihydroisocoumarin derivatives that we identified as dual P-gp and BCRP inhibitors with moderate activity might serve as a promising starting point for further development of novel inhibitors for overcoming cancer MDR. In future work, it will be important to identify additional substituents at other positions of the molecule to significantly improve the potency of the current chemical lead structure.

In a first initial screening ***(R)*-19**, ***(S)*-19**, **16** and **16a** were identified as potential inhibitors of the yeast ABC transporter Pdr5. With these four compounds IC_50_ measurements with respect to R6G transport as well as the ATPase activity were performed. In the case of the transport assay the highest IC_50_ value was determined for ***(R)*-19** with 26.0 ± 3.5 μM. Interestingly the lowest IC_50_ was detected for its enantiomer ***(S)*-19** with 5.3 ± 0.6 μM. The transport IC_50_ values for **16** and **16a** were nearly similar with 15.1 ± 2.1 and 14.6 ± 3.9 μM, respectively. For the ATPase activity it was not possible to reliably determine any IC_50_ value because of the limited solubility of the compounds. The highest ATPase activity inhibition of Pdr5 wild-type was detected for ***(S)*-19** with an inhibition down to 17.3% activity in comparison to Pdr5 WT without any compound. For ***(R)*-19** the ATPase activity was inhibited to 21.5% and for **16** and **16a** the activity was inhibited to 35.4 and 22.8%, respectively. Our data furthermore suggest that these four coumarins are substrates of Pdr5, which are transported in competition to the fluorophore R6G and become inhibitory at higher concentrations, a phenomena often observed for P-gp (Al-Shawi et al., 2003) and Pdr5 (Ernst et al., 2008). In contrast to P-gp, it is obviously not important if there is a hydroxy or methoxy group at position 6, but it seems important that a longer hydrophobic side chain is present at position 3. The two natural substrates 6-methoxymellein (**3)** and angelicoin (**4)** with only a small methyl group at position 3 or compound **14** with no alkyl side chain at all displayed no effect on neither transport nor ATPase activity. Furthermore, in comparison to that the two compounds with a longer alkyl side chain with a hydrophilic group (***(R)*-19a** and ***(S)*-19a**) showed only inhibition to around 40%. In addition to transporter inhibition, the cytotoxic activity of the test compounds was analyzed in sensitive A549 lung carcinoma cells as well as resistant HCT-15 colon carcinoma, H69AR lung carcinoma and MCF-7/MX breast carcinoma cells. Most compounds did not exhibit any toxic effects in cancer cells. One exception was derivative **16a**, which proved to be selectively cytotoxic in BCRP-expressing MCF-7/MX cells with an IC_50_ value of 10.6 μM, but not in P-gp or MRP1-expressing cells. In sensitive A549, cytotoxicity was significantly lower with an IC_50_ value of 53.6 μM. At this point, it would be interesting to further characterize if compound **16a** specifically kills multidrug-resistant cells by analyzing its activity in parental, sensitive MCF-7 cells. If this is the case, gaining insights into the mechanism of action and target would be helpful to find out more about collateral sensitivity in BCRP-overexpressing cancer cells and how to exploit this feature in tumor therapy (Pluchino et al., 2012; Rao et al., 2014).

## Author Contributions

JS, KD, and MB performed experiments, analyzed data, and wrote the manuscript. AW synthesized test compounds. EF and AK provided ellagic acid. NT, LS, HG, JP, EF, and AK contributed to study conception and design and received funding. All authors revised the manuscript.

## Conflict of Interest Statement

EF and AK were employed by company MicroCombiChem GmbH. The remaining authors declare that the research was conducted in the absence of any commercial or financial relationships that could be construed as a potential conflict of interest.

## Footnotes

1 https://www.schrodinger.com/citations
